# Supplementary material for: Adapting population health interventions for new contexts: qualitative interviews understanding the experiences, practices and challenges of researchers, funders and journal editors
Source: BMJ Open. 2022 Oct 26;12(10):e066451. doi: 10.1136/bmjopen-2022-066451 (PMC9615984; doi:10.1136/bmjopen-2022-066451)
Supplement: Author's manuscript [file bmjopen-2022-066451.draft_revisions.pdf]

BMJ Open

Adapting population health interventions for new contexts: Qualitative interviews understanding the experiences, practices and challenges of researchers, funders and journal editors

Journal:	*BMJ Open*
Manuscript ID	bmjopen-2022-066451
Article Type:	Original research
Date Submitted by the Author:	07-Jul-2022
Complete List of Authors:	Copeland, Lauren; Cardiff University, Centre for Development, Evaluation, Complexity and Implementation in Public Health improvement (DECIPHer)<br>Littlecott, Hannah; Cardiff University, Centre for Development, Evaluation, Complexity and Implementation in Public Health improvement (DECIPHer); LMU, Pettenkofer School of Public Health (PSPH), Institute for Medical Information Processing, Biometry and Epidemiology<br>Couturiaux, Danielle; Cardiff University, Centre for Development, Evaluation, Complexity and Implementation in Public Health improvement (DECIPHer)<br>Hoddinott, Pat; University of Stirling, Nursing, Midwifery and Allied Health Professional Research Unit<br>Segrott, Jeremy; Cardiff University, Centre for Trials Research; Cardiff University, Development, Evaluation, Complexity and Implementation in Public Health improvement (DECIPHer)<br>Murphy, Simon; Cardiff University, Centre for Development, Evaluation, Complexity and Implementation in Public Health improvement (DECIPHer)<br>Moore, Graham; Cardiff University, Centre for Development, Evaluation, Complexity and Implementation in Public Health improvement (DECIPHer)<br>Evans, Rhiannon; Cardiff University, Centre for Development, Evaluation, Complexity and Implementation in Public Health improvement (DECIPHer)
Keywords:	Public health < INFECTIOUS DISEASES, PUBLIC HEALTH, QUALITATIVE RESEARCH

SCHOLARONE™
Manuscripts

I, the Submitting Author has the right to grant and does grant on behalf of all authors of the Work (as defined in the below author licence), an exclusive licence and/or a non-exclusive licence for contributions from authors who are: i) UK Crown employees; ii) where BMJ has agreed a CC-BY licence shall apply, and/or iii) in accordance with the terms applicable for US Federal Government officers or employees acting as part of their official duties; on a worldwide, perpetual, irrevocable, royalty-free basis to BMJ Publishing Group Ltd ("BMJ") its licensees and where the relevant Journal is co-owned by BMJ to the co-owners of the Journal, to publish the Work in this journal and any other BMJ products and to exploit all rights, as set out in our licence.

The Submitting Author accepts and understands that any supply made under these terms is made by BMJ to the Submitting Author unless you are acting as an employee on behalf of your employer or a postgraduate student of an affiliated institution which is paying any applicable article publishing charge ("APC") for Open Access articles. Where the Submitting Author wishes to make the Work available on an Open Access basis (and intends to pay the relevant APC), the terms of reuse of such Open Access shall be governed by a Creative Commons licence – details of these licences and which Creative Commons licence will apply to this Work are set out in our licence referred to above.

Other than as permitted in any relevant BMJ Author's Self Archiving Policies, I confirm this Work has not been accepted for publication elsewhere, is not being considered for publication elsewhere and does not duplicate material already published. I confirm all authors consent to publication of this Work and authorise the granting of this licence.

For peer review only

Adapting population health interventions for new contexts: Qualitative interviews understanding the experiences, practices and challenges of researchers, funders and journal editors

Lauren Copeland[1], Hannah Littlecott[1,2], Danielle Couturiaux[1], Pat Hoddinott[3], Jeremy Segrott[4], Simon Murphy[1], Graham Moore[1], Rhiannon Evans[1]

1 Centre for Development, Evaluation, Complexity and Implementation in Public Health improvement (DECIPHer), School of Social Sciences, Cardiff University, Wales, UK

[2] Pettenkofer School of Public Health (PSPH), Institute for Medical Information Processing, Biometry and Epidemiology (IBE), LMU Munich, Elisabeth-Winterhalter-Weg 6, 81377, Munich, Germany

3 Nursing Midwifery and Allied Health Professions Research Unit, University of Stirling, Scotland, UK

4 Centre for Development, Evaluation, Complexity and Implementation in Public Health improvement (DECIPHer), Centre for Trials Research, Cardiff University, Wales, UK

Corresponding author: Dr Lauren Copeland (copelandlc@cardiff.ac.uk).

Word count: 5588

Acknowledgments

We thank the ADAPT Study management team which in addition to co-authors included Dr Mhairi Campbell, Prof Peter Craig, Dr Ani Movsisyan, Prof Alicia O'Cathain, Dr Lisa Pfadenhauer, Dr Eva Rehfuess, Dr Britt Hallingberg, Dr Laura Arnold, and Prof Laurence Moore. also thank our advisory group which included Prof Penelope Hawe, Prof Frank Kee, Dr Andrew Booth, Prof Frances Gardner and Dr Julie Bishop. We also thank anonymous members of our qualitative interviewees.

Key Words: Adaptation, Public health, Practical challenges, Guidance

For peer review only

ABSTRACT

OBJECTIVES: Research on the adaptation of population health interventions' for implementation in new contexts is rapidly expanding. This has been accompanied by a recent increase in the number of frameworks and guidance to support adaptation processes. Nevertheless, there remains limited exploration of the real-world experiences of undertaking intervention adaptation, notably the challenges encountered by different groups of stakeholders, and how these are managed. Understanding experiences is imperative in ensuring that guidance to support adaptation has practical utility. This qualitative study examines researcher and stakeholder experiences of funding, conducting and reporting adaptation research.

SETTING: Adaptation studies.

PARTICIPANTS: Participants/cases were purposefully sampled based to represent a range of adapted interventions, types of evaluations, expertise, and countries. Semi-structured interviews were conducted with a sample of researchers (n=23); representatives from research funding panels (n=6); journal editors (n=5) and practitioners (n=3).

MEASURES: A case study research design was used. Data were analysed using the Framework approach. Overarching themes were discussed within the study team, with further iterative refinement of sub-themes.

RESULTS: The results generated four central themes. The first three relate to the experience of intervention adaptation 1) involving stakeholders throughout the adaptation process and how to integrate the evidence base with experience; 2) selecting the intervention and negotiating the mismatch between the original and the new context; and 3) the complexity and uncertainty when deciding the re-evaluation process. The final theme (4) reflects on participants' experiences of using adaptation frameworks in practice, considering recommendations for future guidance development and refinement.

CONCLUSION: This study highlights the range of complexities and challenges experienced in funding, conducting and reporting research on intervention adaptation. Moving forward, guidance can be helpful in systematising processes, provided that it remains responsive to local contexts and encourage innovative practice.

- **Strengths and limitations of this study**
- The methodology captured a diverse and nuanced range of perspectives in relation to intervention adaptation.
- The sampling ensured that we captured a wide range of studies including micro, meso and macro level interventions which allowed us to explore adaptation research experiences.
- The primary limitation of this study was that we were unable to recruit Patient and Public Involvement (PPI) and policy makers, limiting diversity in the perspectives reflected in our data

For peer review only

- Without the input from policymakers and PPI the study lacks insight into how intervention adaptation is commissioned and resourced at a national and local level and how adaptation is understood by PPI contributors.

For peer review only

1.0 Background

Research on the adaptation of population health interventions' for implementation in new contexts is evolving at speed[1-7]. Adaptation is when intentional changes are made to an evidence informed intervention, either pro-actively or in response to emerging challenges, in order to improve the contextual fit within a new setting. This evolution accompanies the increased recognition that intervention effects do not always directly transfer to new contexts[4 5 8-10] and that adapting an existing intervention may be more efficient than de novo intervention development[11]. Within the ADAPT study population health interventions are defined as interventions or policies in public health or health services that aim to change the population distribution of risk at either the micro, meso or macro level[12].

In response to the emerging research on adaptation there has been a significant increase in frameworks and guidance to support these processes [1 2 13-15]. While a number of these frameworks are explicitly grounded in empirical examples of adaptations, they often provide limited, exploration of the real world practice of undertaking adaptation, notably the complexity and challenges encountered by a diverse range of stakeholder groups [16]. Equally, stakeholder involvement and co-production has been increasingly recognised as imperative in the complex process of development, adaptation and evaluation of interventions [14 17 18]. This, however, is something that has been underexamined in relation to adaptation. Furthermore, there has been limited research exploring the uptake and usefulness of guidance and frameworks to support adaptation which is important given that it seems to be rarely used. Publication of existing guidance has been relatively recent, which may explain the limited reports of guidance use and impact. It is important to consider how frameworks have been, and might be, integrated into real world practice to maximise their impact[19].

This qualitative study examines stakeholders' experiences of funding, conducting and reporting of adaptation of complex interventions. It aims to understand the complexities and the practical challenges of conducting adaptation research. It was undertaken concurrently with other work packages as part of the ADAPT study (2018-2020), which aimed to develop evidence and consensus-informed guidance[12] that was grounded in the theoretical, methodological and real-world understandings, experiences, and perspectives of a diverse range of relevant stakeholders.

1.1 The ADAPT Study

The ADAPT study (2018-2020) was funded by the UK MRC-NIHR methods panel to develop population health interventions' adaptation guidance [7]. It aims to support researchers, policy-makers, practitioners, funders and journal editors in the funding, conduct and reporting of research on adaptation. The ADAPT study comprised of three work packages: 1) A systematic review of existing adaptation guidance[2] and scoping review of case examples of intervention adaptation[20] ; 2) A qualitive study using semi-structured interviews to explore the understandings, perspectives and experiences of researchers, funders, journal editors, and policy and practice stakeholders; and 3) A Delphi expertise consensus exercise to scope the clarity of the definitions and constructs used in the guidance, explore and capture key debates, identify agreement on important adaptation processes, and ascertain areas where there is limited consensus [21]. These work packages formed part of the process to develop the guidance and the current study forms part of work package 2.

For peer review only

2.0 Methods

This paper reports on the semi-structured interviews which were undertaken between April and September 2019 concurrently to inform the ADAPT study guidance. Participants were stakeholders with experience of intervention adaptation. Ethical approval was provided by Cardiff University's School of Social Sciences Ethics Committee (Ref: SREC/3165).

A case study research design was used in the first instance [22-24]. A case of adaptation was defined as a population health intervention that had previously been subjected to adaptation or was currently being adapted. For each of the cases, we aimed to interview a researcher involved in intervention adaptation and/or re-evaluation, Patient and Public Involvement (PPI) contributors who were part of the intervention adaptation and where possible an associated decision-makers (e.g. policy and/or practitioner stakeholder) who may have had experience of implementing the intervention in the new context. Although, in many cases, there was only one perspective represented per case. Funders and journal editors were not linked to specific cases but contributed to understanding of the wider evaluation context. As the study unfolded it became increasingly challenging to recruit multiple and varied participants per case. Therefore, in many cases, there was only one perspective represented per case. In order to redress this shortcoming, more emphasis was placed on exploring diverse perspectives across different participants linked to different adapted interventions rather than comparing across cases.

2.1 Recruitment and sampling

Researchers, policy-makers and practitioners were initially identified through case examples of adapted interventions retrieved as part of the ADAPT systematic review[2] and scoping review[20]. The studies were included if they were a primary study describing an adaptation process and/or an evaluation of an evidence-informed intervention adapted to a new context, focused on public health and/or health service interventions, and were published from 2000 onwards. Studies were excluded if the intervention had been designed de novo for a specific context or examined clinical procedures, such as surgery. The 312 retrieved interventions were classified according to: the socio-ecological domain where the theory of change primarily operated (mico, meso or macro); the contexts between which the intervention was transferred (e.g. country to country or population to population within a country); study design (e.g. effectiveness or feasibility); and outcomes (i.e., favourable or unfavourable). The purpose of this was to achieve insight into variations in the nature of system disruptions (areas the intervention intends to target and enact change upon), adaptations and adaptations processes and how they might explain different outcomes. During the recruitment process participants were emailed the information sheet and the consent form and asked to provide consent to take part in the study prior to the interview. All participants were given at least a week to consider their participation prior to their completion of the consent form.

All 23 primary researchers, who were recruited, were contacted, with the aim to snowball sample further stakeholders. This was largely ineffectual, this yielded 3 participants due to the age of some of the studies, therefore, additional recruitment strategies were used: expertise recommendation; advertising through the

For peer review only - http://bmjopen.bmj.com/site/about/guidelines.xhtml

Involving People charity, which supports public and patient involvement in research; and Twitter promotion targeting the European Society for Prevention Research and the Society for Prevention Research. Funders were identified from international funding boards. Journal editors were identified from the relevant journals that published the case examples of adapted interventions.

A total of 37 participants were recruited to the study. The sample comprised of 23 researchers involved in the adaptation of 23 interventions (cases) (Table 1). The researcher participants conducted their work in the United States of America (US) (n=12), United Kingdom (UK) (n=2), New Zealand (n=2), India (n=1), France(n=1), Germany(n=1), Spain(n=1), Italy(n=1), China (n=1), German (n=1). Of the three practitioners, one practitioner was linked to one of the 23 interventions and two were recruited via expert recommendation. These practitioners had experience of adapting interventions for addictions b these interventions were not one of the 23 interventions included. Two of the practitioners conducted their work within the UK and one conducted their work in France. The study did not succeed in recruiting PPI representatives or policymakers. Six representatives from funding panels participated. They were based in the USA (n=1), UK (n=3), Germany (n=1), or had an international remit (n=1). The 5 journal editors represented global health (2) or public health (3). Their primary publishing location was US (2), Canada (1), countries across Europe (1) and Australia (1). Amongst the approached individuals that did not take part, invitees stated that the subject matter was not relevant to them (6), their workload was too high, (2) or they did not respond after 3 follow-up contacts (64).

2.2 Data collection

Interviews were conducted by two members of the research team (LC/HL). Tailored topic guides were developed for each set of researchers and stakeholders, informed by the study research questions and emerging data from the systematic[2] and scoping reviews[20]. Guides were refined and confirmed with the wider study team prior to data collection. They were also reviewed as the interview progressed and no revisions were necessary. They considered: the definition of intervention adaptation and related concepts; experiences of undertaking adaptation and re-evaluation, in addition to funding and reporting adaptation processes; and views on adaptation guidance development (see appendix A). Interview length ranged from 40 to 75 minutes and were conducted via telephone or Skype. Interviews were audio-recorded and transcribed verbatim by a professional transcription company. Transcripts were reviewed for accuracy and anonymised.

2.3 Data analysis

Four members of the research team (LC; DC; HL; RE) analysed the data using the Framework approach[25]. The three participant data sets (researchers and practitioners; funders; journal editors) were treated separately. Three different coding frameworks were then developed by the four researchers, using two interviews from each data set which were randomly chosen. Each framework included both a priori codes and in vivo codes. The remaining data were coded by a single researcher. The frameworks evolved during analysis, with the new codes discussed and confirmed by the team, before being applied to previously coded data. To ensure reliability, 10% of the data was independently checked by a second researcher (RE/ DC). Disagreements between researchers were resolved through discussion. NVivo 10 supported data analysis and storage.

For peer review only

The four researchers charted coded data into the three separate framework matrixes. Data within and across the matrixes were compared and contrasted by two members of the research team (LC; RE) as part of the interpretative process of generating themes. To aid this process, visual maps were created. We created five over-arching themes, each with a set of related subthemes: adaptation decision-making and processes, re-evaluation decision-making and processes; funding; publication; and recommendations for adaptation guidance. Overarching themes were presented to the wider ADAPT study team who suggested further refinements of subthemes. As the ADAPT Delphi consensus exercise progressed and areas of consensus and disagreement emerged, we undertook additional analysis of the qualitative data to bring insight to these emerging perspectives.

2.4 Patient and public involvement

This research was conducted without patient involvement. We involved policy and practice representatives with experience of intervention adaptation in qualitative interviews and our study advisory group.

2.5 Reflexivity

LC and HL conducted the interviews and LC, HL, DC and RE conducted the data analysis. At the time of analysis, LC and HL were Research Associates with PhDs. DC was a Research Assistant with an MSc. RE was a Senior Lecturer with a PhD. All are experienced qualitative researchers who have received training in conducting interviews and thematic and framework analysis. None of the researchers apart from RE and HL had a prior relationship before the study. RE and HL had worked previously on studies together. The participants did not know the researchers prior to the study. The participants understood the researchers were conducting the interviews as part of the ADAPT study in order to explore their experiences of conducting adaptation studies. RE and HL have a methodological expertise in adaptation which may have influenced their interview style and analysis of the data based on their extensive prior knowledge of the area. LC and DC were new to adaptation, but both have worked on process evaluations looking at context. Therefore, their focus on context may have influenced the interview style and analysis. The interviews were guided by topic guides developed by the wider team which will have negated some of the researcher bias. Ten percent of the analysis was double coded to negate some of the bias of the researchers.

For peer review only

Table 1: Adaptation Cases Sample Characteristics

Stage of Study	Participant (researcher/ practitioner)	Type of Intervention (macro/meso/micro)	Research Design (feasibility study or randomised control trial (RCT))	Target of Intervention	Contextual Transfer (country to country/ population to population/ setting to setting)	Evaluation Outcome
**Adaptation cases with 2 stakeholder perspectives**						
Completed	Researcher and Practitioner	Meso	Feasibility	Diet and exercise	Policy to different settings	Infeasible
**Adaptation cases with 1 stakeholder perspective**						
Completed	Researcher	Macro	Feasibility	Reproductive and child health	Country to country	Feasible
Completed	Researcher	Macro	Feasibility	Road traffic injury	Country to country	Feasible
Completed	Researcher	Meso	RCT	Addictions	Country to country	Effective
Completed	Researcher	Meso	Feasibility	Sexual health	Population to population	Feasible
Completed	Researcher	Meso	Feasibility	Sexual health	Population to population	Effective
Completed	Researcher	Meso	Feasibility	Hearing	Setting to setting	Feasible
Completed	Researcher	Micro	RCT	Parenting	Country to country	Effective
Completed	Researcher	Micro	RCT	Weight Loss	Population to population	Effective
Completed	Researcher	Micro	Feasibility	Diabetes prevention and management	Population to population	Feasible
Completed	Researcher	Micro	Feasibility	Smoking: cessation	Population to population	Feasible
Completed	Researcher	Micro	Feasibility	Mental health	Country to country	Feasible
Completed	Researcher	Micro	Feasibility	Childhood obesity	Setting to setting	Feasible

For peer review only

Completed	Researcher	Micro	Feasibility	Exercise	Population to population	Infeasible
Completed	Practitioner	Micro	Feasibility and RCT	Addictions	Setting to setting	Mixed
Completed	Practitioner	Micro	Feasibility and RCT	Addictions	Setting to setting	Mixed
In progress	Researcher	Meso	RCT	Lung health	Country to country	N/A
In progress	Researcher	Meso	RCT	Cancer	Population to population	N/A
In progress	Researcher	Meso	Feasibility	Weight loss	Country to country	N/A
In progress	Researcher	Micro	RCT	Diabetes prevention and management	Population to population	N/A
In progress	Researcher	Micro	RCT	Diabetes prevention and management	Population to population	N/A
In progress	Researcher	Micro	RCT	Diabetes prevention and management	Population to population	N/A
In progress	Researcher	Micro	Feasibility	Weight loss	Country to country	N/A
In progress	Researcher	Micro	Feasibility and RCT	Diet and exercise	Country to country	N/A

For peer review only

3.0 Results

The analysis generated four central themes. The first three relate to participants' experiences of and reflections on intervention adaptation 1) experience of involving stakeholders in the adaptation process; 2) negotiating the mismatch between the original context where the intervention was delivered and the new context; 3) deciding upon the re-evaluation process. The final theme (4) reflects on participants' experiences of using adaptation frameworks, and their recommendations for future guidance development.

3.1 Involving stakeholders

Participants foregrounded the importance of involving a diverse range of stakeholders (intervention developer, industry policy makers, implementers and organisations supporting delivery, and participants) throughout the adaptation process. The reasons for stakeholders' engagement were primarily related to them having more knowledge of how the intervention functions or the characteristics of the new context, compared to those leading the process, who were often academics.

> *"Absolutely I mean Apple doesn't develop an iPhone without doing market research. We, as researchers and clinicians and doctors, you know, we have the knowledge from the textbook and from the theory and everything we've learnt, but we don't understand how to apply to a specific population without their knowledge and their expertise to teach us how it would be relevant for them." P008 researcher micro in progress*

Stakeholder involvement was considered to be so imperative within the adaptation process, that funding representatives maintained that it was a central criterion applied by an assessment panel:

> *"they will always come together if there are key methodological flaws … that will come out very quickly and that includes things like the PPI involvement is simply not there, and that is something that they consider mission critical, and as I say." P004 Funder*

The practicalities of involving stakeholders was also noted as problematic. This could be due to potential conflicts of interest between stakeholders and researchers. For example, stakeholders could direct adaptations in ways that are under-researched. One practitioner discussed their experiences of working in a setting in which they felt the evidence base did not work within the context creating a conflict between experience and evidence driven adaptations:

> *"So although it's sitting there saying this is the evidence base, you should be doing this, it just categorically doesn't work in our setting. So that's an example of how if you just applied the evidence base it just, it would be hopeless." P022 Practitioner*

For peer review only

This causes a conflict between ensuring that all contributions are supported by evidence i.e. low risk of bias vs. changes being made based on stakeholder experience of the setting. This can cause issues when reviewers are reviewing the study and assessing how the adaptations have been justified. For example, one journal editor highlighted that many reviewers are not experts in adaptation and do not always assess the quality of PPI involvement:

> *", there's a lot more need for PPI stuff, there's a lot more need for doing more of the background work I'd say, the formative work to get input from key stakeholders and recipients… and that's the kind of stuff that a lot of reviewers don't even pick up or think about because they don't do the work." P003 Journal editor*

In addition, many stakeholders were noted as making contributions at different times across the adaptation process, often with differing opinions and different expressions of need. This made it difficult to undertake adaptation systematically, incorporating and balancing the 'stylistic differences' across the stakeholders about what should be done:

> *"But through just team discussion and supervision and peer supervision, we definitely have a kind of, if you like a general consensus that adaptations are necessary, and I think we all do that. But it's just the degree to which we do it, and exactly how we do it will vary from clinician to clinician." P022 Practitioner*

Stakeholder involvement with adaptation, as described by all the participants, is key as they can provide insight into how the intervention might function in the new context and what adaptations the new context may require. However, it was noted that there are multiple perspectives and differences of opinion and values about what to adapt and why, which can pose barriers to effective adaptation.

3.2 Selecting the intervention and negotiating the mismatch between the original and the new context

Most data pertaining to participants' experiences of intervention adaptation centred on how to select the right intervention for the new context, how to decide if adaptation is necessary and if so which adaptations to undertake. Overall, there was a sense of a tension between wanting to select the intervention based on evidence vs ensuring it could be delivered with the resources and money available. However, in reality there are competing practical factors that need to be taken into account to guide decisions. For example, one researcher reflected on the issue of balancing the evidence base versus the practical aspect of ensuring that the intervention could be delivered in a low-income country with different resources.

> *"The most important thing I take first and foremost is the degree to which the evidence is available and is robust enough to adapt into different environments and whether it's adaptable. Whether the rigour and the tool's ability of the intervention may be suitable in high income and may not even be adaptable in low and middle income countries." P023 researcher macro feasible*

For peer review only

Again, pragmatically, participants chose to use evidence-based interventions that were already embedded in the country as it had already achieved buy in amongst stakeholders and there were already the mechanisms in place to support delivery. Participants often selected interventions based on an awareness or prior relationships with the developers or evaluators as they had built a trusting and respectful working relationship:

> *"It is probably like most studies, I would love to say it was fully systematic!. … We chose it because they are very faith-based and we thought that would work but to be honest, a good bit of it was that these were two good investigators I knew." P010 researcher micro in progress*

When it came to the process of modifying the intervention, participants reflected on how time-consuming and therefore complex adaptation could be. Some maintained that it could take up to a year to iteratively adapt the intervention, depending on the level of complexity involved. There was a clear sense that the current funding climate, which often subsumed adaptation into early phases of evaluation, did not permit the required time to fully undertake comprehensive adaptation:

> *"it's very rare I guess, to get funding that is explicitly and exclusively for adapting a campaign. So that kind of funding mechanism is unusual, but it really gave us a chance to do things the right way." P016 researcher meso infeasible*

In general, it was reported that there was a limited amount of time that could be funded to conduct the adaptation process. One funder commented that they only allow 6 months for this, which they felt was not sufficient time to conduct the adaptation process.

> "*I mean probably a limitation of the system is that we have kind of a rule in that we'll only fund up to six months of adaptation work.*" P003 *Funder*

Overall, the adaptation process is complex and involves balancing pragmatic decisions with decisions based on evidence, which researchers have been trained to prioritise. It requires time and a systematic approach to ensure a thorough process is undertaken, however, this is difficult if this process is not recognised by the funder and with no consistent and systematic approach to follow, at the time of data collection.

3.3 Deciding upon the re-evaluation process

Some re-evaluation was considered by most to be necessary following the introduction of an adapted intervention into a new context, although deciding upon the nature and extent of new evaluation required was described as challenging. Participants discussed how they considered the utility of different study designs for re-evaluation and the complexity of deciding upon an approach. A number of individuals suggested that feasibility testing, process evaluations and implementation studies are most relevant, given that the most pertinent research questions relate to mechanism of action.

For peer review only

Despite some indication of the rationale for different evaluations designs, in practice participants encountered numerous challenges to the conduct of a scientifically robust evaluation. While it was common for participants to state a preference for less resource intensive evaluation, on occasion they did acknowledge the importance in resolving uncertainty. It appears that this can lead to researchers and funders being at odds as researchers feel they can borrow strength from the existing evidence and skip steps. Whereas, the funder default may be still to expect evaluation as if it is a new and untested intervention.

> *"It was forced upon us, I think it'd be true to say. [Laughs]. We'd decided, obviously, on an adaptation phase and, in fact, we wanted to go straight for a full trial. Because our views, you know, naivety galore, thought that this was a great programme from (name of place 3), and why not de-Anglify it, make some adaptations and pretty much roll straight out into full trial. Obviously, I think we probably had a feasibility, you know, internal pilot, I can't quite remember, actually, at our Stage 1 application. And they came back, saying, "No, no, we don't think you should go beyond feasibility phase."". P014 researcher micro in progress*

Centrally, participants reflected on the resource required for extensive re-evaluation, notably in terms of time and funding, which could not always be acquired:

> *"We need theory building and we need that work, but I feel like with the limited funding that's available, particularly in the (name of place 1) and the drastic health conditions that we have, that we probably should start matching and integrating our efficacy trials with our effectiveness trials, that we develop things with an eye for sustainability and thinking about how to leverage the current resources that we have.." P008 researcher micro in progress*

There are clear challenges to re-evaluation which derive from a lack of certainty about how researchers make decisions about what type of evaluation to undertake and how funders make judgements about what to fund. There are merits to the different research designs however participants did not know which design was most suitable for their intervention and context and, at the time of data collection, there was no recommended systematic approach for how to make decisions about re-evaluating adapted interventions to utilise. Therefore, these findings identified a real need for guidance to inform the current uncertainty surrounding funding decisions and resources.

3.4 Participants' experiences of using adaptation frameworks and recommendations for future guidance

Participants described limited awareness of adaptation frameworks, rarely mentioning their uptake. However, when mentioned they were seen as important to conduct research in a systematic manner. In the absence of dedicated adaptation models, most participants drew upon generic intervention development and evaluation guidance to support their decision-making processes. There were recognised limitations with existing adaptation frameworks and guidance. First, they were considered too long and time consuming to be realistically applied given the resource constraints associated with the current funding climate. One participant followed the Map of

For peer review only

the Adaptation Process[11], which was deemed to be shorter compared to other frameworks, due to time constraints.

> *"Yes, so I ... we followed the Map of the Adaptation Process, right, that is more, it's a shorter version, and it's still grounded in theoretical approaches.... Time was one of the factors, being cost effective was another, and we didn't have enough funding for a thorough and long adaptation process." P012 researcher meso feasible*

Second, participants suggested that guidance can often be too conceptual, making it difficult to implement in real world practice. In particular there was a challenge in applying and tailoring generic, abstract thinking to the detailed specifics of the intervention they were working with:

> *"It's so specific to each intervention, these things are so specific that it's really hard to pin them down, and to say well, to move from the concept to the actual practical side of things is quite difficult. I think that's probably the biggest challenge." P005 researcher meso unfeasible*

Reflecting on these issues, participants expressed a number of recommendations for the development or future refinement of adaptation guidance. Some participants expressed a need for an overarching, systematic timeline of adaptation phases and re-evaluation approaches to allow for a common understanding across stakeholders of the adaptation and re-evaluation process.:

> *"I think it's always good to have a systematic kind of timeline in terms of when you should do stuff." P019 researcher meso in progress*

In order to fully recognise the value of stakeholder involvement, participants stated that guidance also needed to target the full range of relevant stakeholders This can enhance buy in, by ensuring that the guidance can be understood by different stakeholders and providing a process for how to involve them throughout the study:

> *"....adaptation requires time and results and skills, and policymakers don't know that (laughs) at all. I think it's important to just have some guidelines or tools to let them understand, because I'd rather that it's one of your targets, but I think that's also the information you should give that's different for policymakers or practitioners or researchers." P017 researcher meso effective*

Finally, there was suggestion for a checklist in terms of what to include when reporting adaptation processes in papers for publication. Participants talked about multiple influences in terms of publications suc[26]h as the time the researcher has, the type of paper that gets published and the need to accurately report the adaptation process.

> *"So having like a very big and broad checklist of things to think about, and probably will be something that you have nothing to do with you, but at least you can follow that one, like a third guideline to see what other things that you need to report." P001 researcher micro feasible*

This will aid publication of adaptation process papers as well as outcome papers.

For peer review only

3.0 Discussion

This qualitative study explored the real-world experiences of researchers, practitioners, funders and journal editors of conducting adaptation research. This work has highlighted a number of key challenges; 1) involving stakeholders throughout the adaptation process and how to integrate the evidence base with experience; 2) selecting the intervention and negotiating any incongruence between the original intervention and the new context; 3) the complexity and uncertainty of deciding upon the re-evaluation process; and (4) participants' experiences of using adaptation frameworks in practice. These findings contributed to the ADAPT guidance[12] and address important gaps in our knowledge about the adaptation, implementation and re-evaluation of complex interventions in new contexts.

The participants repeatedly highlighted the importance of stakeholder involvement throughout the adaptation and re-evaluation process[14 17] as they provided an insight into the intervention's functioning or the features of the new context. However, there are challenges in co-production research[18], as raised by the participants, in terms of ensuring adaptation is conducted in a systematic and evidence-based manner. This uncertainty is echoed in the work of community-based participatory research in which it is challenging to anchor it in comprehensive theoretical framework[27]. Due to the importance of stakeholders, the participants stressed the need for the guidance to be accessible and presented in a way that helps to involve stakeholders.

Selecting the intervention and negotiating the mismatch between the original and the new context presented challenges for the participants. They reflected that the selection process was complicated as researchers wanted to base their decisions on the current evidence base, however, they also acknowledged that there were practical considerations that could compete with the evidence base or override it. For example, after reviewing all the evidence on an intervention, one might find that the evidence indicates a particular intervention is the most effective. However, it may be too resource intensive to be implemented within the new context[28]. Therefore, pragmatically it might be best to select an intervention that is already embedded in the country as it already has the mechanisms in place to support delivery and in addition has gained the buy in of stakeholders[29]. This balance between evidence and practice-based decisions is a consistent challenge throughout public health research and is an aspect that needs to be resolved to help bridge the gap between research and practice [30-32]. This is an unresolved area which, if left unaddressed, could impact the scientific merit of the selected intervention[32]. Therefore, guidance is needed to clarify the intervention selection process and bridge the research and practice gap.

Participants reported the re-evaluation process and the merits of different research designs. Overall, it was found that there is currently much uncertainty as to which design to choose. Researchers reported deciding that more extensive adaptation required a RCT (it was acknowledged that this design might not always be appropriate to assess the intervention e.g a policy intervention at a macro level) to be conducted as there was greater uncertainty as to whether the intervention would remain effective. They also indicated that if the original intervention has had multiple RCTs already conducted showing effectiveness in the original context, they perceived that no pilot would be required during revaluation[26 33]. However, funders have recommended to researchers that pilot studies should be conducted as an initial re-evaluation stage[34 35]. Participants felt that there

For peer review only

was a tension between these time requirements and the funding climate, at the time of data collection, which did not accommodate the required time to fully undertake comprehensive adaptation. Given the current funding climate and time to test feasibility and effectiveness, participants expressed a need for less resource intensive evaluation[34]. To address this issue in part it is important to place value on the information already existing for the intervention in its original context. This can aid decisions on whether a full evaluation is warranted prior to implementation[36].

Overall participants reflected that there were several challenges of using adaptation frameworks in practice. A number of adaptation frameworks have been developed in order to provide some guidance for this emerging field. However, while some aspects of good practice are clear, there are still areas on which there is no consensus on best practice[2]. Some frameworks were reported to be difficult to implement within real world settings due to the oversimplified, list-like format which does not reflect the complex nature of the adaptation process[37]. Further to this it was reported some frameworks were too time consuming, leading to interpretation issues due to funding restrictions. Participants expressed a desire for guidance to take into account real world challenges and for it to reflect the different time and funding availability.

There are practical challenges that have been raised by the participants within this study. This area is constantly progressing with emerging adaptation frameworks [1,2,13,14] and now with the recently published ADAPT guidance there is a need to assess how such guidance can help support these identified practical challenges going forward. As highlighted by the participants, there are limited resources and funding available, as well as a drive towards value for money. Therefore, adaptation can provide a cost-effective way of tackling the health needs of different settings[11], with the right support and buy-in from funding organisations. Overall, there was a clearly expressed need for guidance from study participants. However, in this quickly evolving field, it is important to engage with how the guidance is being used and the nuance and diversity in perspectives on an ongoing basis.

3.1 Study Strengths and Limitations

The primary limitation of this study was that the diversity of perspectives reflected in our data was limited by failure to recruit from some target groups. Although there were multiple attempts to recruit PPI and policy makers, we were unsuccessful., therefore in the process of developing case studies. We suspect that this issue with recruitment was due to the majority of the studies being completed, therefore, many of those involved in the study had moved onto other job so were uncontactable or did not have the time to take part. This is reflective of the nature of research culture in which people are contracted only for the duration of the project. We were unable to recruit policymakers after reaching out to a number of contacts due to the busy nature of their jobs. As such their perspective, which may contrast with the generated data, were not included. In addition, without the input from policymakers the study lacks insight into how intervention adaptation is commissioned and resourced at a national and local level. Furthermore, while we aimed to sample people involved in a wide range of interventions, operating across the micro, meso, and macro domains, we were only able to identify two macro-level interventions meeting our criteria for adaptation[20]. This may be a consequence of such interventions, notably national policies, not being explicitly framed as adaptations even when derived from principles and practices that are implemented elsewhere. Regardless of these limitations, the data did capture a

For peer review only - http://bmjopen.bmj.com/site/about/guidelines.xhtml

diverse and nuanced range of perspectives in relation to intervention adaptation. It provided complementary data that contributed to and triangulated with the other ADAPT work packages and facilitated the production of comprehensive guidance for researchers on adaptation[38].

3.2 Practice implications

As a result of this study there are a number of recommendations for conducting adaptation research. Participants identified that a systematic approach to adaptation and a checklist for publication was vital to ensure the intervention and its interaction with the context are adequately considered, while directing available resources to the most important areas of uncertainty, and that all pro-active and responsive adaptations are captured and justified both pre and post adaptation (researcher or practitioner led)[39-41]. However, as this is a new and developing field, there is also a need for flexibility to allow for innovation within the field. It is also important for the adaptation process to be accessible and work for different stakeholders to ensure their involvement throughout.

3.3 Conclusions

This study highlights the range of challenges experienced in funding, conducting and reporting research on intervention adaptation. This is partly due to uncertainty about the processes that should be undertaken, and the fact that, at the time of study conduct, frameworks to support adaptation have only recently emerged. Moving forward, guidance on intervention adaptation, including the ADAPT guidance, may be helpful in systematising processes provided that they remain responsive to the local contexts. Therefore, there is a need to assess if the current ADAPT guidance, whose development was informed by the results of this study and published after data collection and analysis for this study took place, can provide clarity. There is also a need to assess and ensure that this guidance is not being too reductionist, as this is an emerging area which requires room to grow[41]. Future research to monitor how adaptation research evolves, particularly as the ADAPT guidance begins to be used in real world practice, would improve knowledge and understanding. This learning will help to support further development and refinement of the guidance, ensuring that future iterations are responsive to the everchanging context of evaluation research.

Data availability: No additional data available

Funding statement: The ADAPT Study was funded by the MRC-NIHR Methodology Research Programme [MR/R013357/1]. The project was undertaken with the support of The Centre for the Development and Evaluation of Complex Interventions for Public Health Improvement (DECIPHer), a UKCRC Public Health Research Centre of Excellence. Joint funding (MR/KO232331/1) from the British Heart Foundation, Cancer Research UK, Economic and Social Research Council, Medical Research Council, the Welsh Government, and the Wellcome Trust, under the auspices of the UK Clinical Research Collaboration, is gratefully acknowledged. The study was also supported by its successor, the Centre for Development, Evaluation, Complexity and Implementation in Public Health improvement, funded by Health and Care Research Wales from 2020. Peter Craig and Mhairi Campbell receive funding from the UK Medical Research Council (MC_UU_12017-13) and the Scottish Government Chief Scientist Office (SPHSU13).

For peer review only - http://bmjopen.bmj.com/site/about/guidelines.xhtml

Competing interests

All authors have completed the ICMJE disclosure form (available on request from the corresponding author) and declare the funding as described above to support the work. The authors declare no financial relationships with any organisations that might have an interest in the submitted work in the previous three years, and no other relationships or activities that could appear to have influenced the submitted work.

Author Contributions: GM, PH, SM, and JS: Conceptualisation, funding acquisition, and writing- review & editing

RE: Conceptualisation, funding acquisition, formal analysis, methodology, project administration, supervision, visualization, writing-original draft preparation and writing-review & editing.

LC: Data curation, formal analysis, project administration, visualization, writing-original draft preparation and writing-review & editing.

HL: Conceptualisation, funding acquisition, data curation, formal analysis, and writing-review & editing

DC: Data curation, formal analysis, and writing-review & editing.

Statement of independence of researchers from funders

The funders had no role in the conduct of the research or publication of its findings.

For peer review only

References

1. Escoffery C, Lebow-Skelley E, Haardoerfer R, et al. A systematic review of adaptations of evidence-based public health interventions globally. *Implementation Science* 2018;13(1):125.
2. Movsisyan A, Arnold L, Evans R, et al. Adapting evidence-informed complex population health interventions for new contexts: a systematic review of guidance. *Implementation Science* 2019;14(1):105.
3. Howarth E, Devers K, Moore G, et al. Contextual issues and qualitative research. Challenges, solutions and future directions in the evaluation of service innovations in health care and public health: NIHR Journals Library 2016.
4. Craig P, Di Ruggiero E, Frolich KL, et al. Taking account of context in population health intervention research: guidance for producers, users and funders of research. 2018
5. Pfadenhauer LM, Gerhardus A, Mozygemba K, et al. Making sense of complexity in context and implementation: the Context and Implementation of Complex Interventions (CICI) framework. *Implementation science* 2017;12(1):21.
6. Pfadenhauer LM, Mozygemba K, Gerhardus A, et al. Context and implementation: a concept analysis towards conceptual maturity. *Zeitschrift für Evidenz, Fortbildung und Qualität im Gesundheitswesen* 2015;109(2):103-14.
7. Evans RE, Moore G, Movsisyan A, et al. How can we adapt complex population health interventions for new contexts? Progressing debates and research priorities. *J Epidemiol Community Health* 2020
8. Evans RE, Craig P, Hoddinott P, et al. When and how do 'effective'interventions need to be adapted and/or re-evaluated in new contexts? The need for guidance: BMJ Publishing Group Ltd, 2019.
9. Hawe P, Bond L, Butler H. Knowledge theories can inform evaluation practice: what can a complexity lens add? *New Directions for Evaluation* 2009;2009(124):89-100.
10. Cambon L, Minary L, Ridde V, et al. Transferability of interventions in health education: a review. *BMC public health* 2012;12(1):497.
11. McKleroy VS, Galbraith JS, Cummings B, et al. Adapting evidence–based behavioral interventions for new settings and target populations. *AIDS Education & Prevention* 2006;18(supp):59-73.
12. Moore G, Campbell M, Copeland L, et al. Adapting interventions to new contexts—the ADAPT guidance. *bmj* 2021;374
13. Stirman SW, Miller CJ, Toder K, et al. Development of a framework and coding system for modifications and adaptations of evidence-based interventions. *Implementation Science* 2013;8(1):65.
14. Kirk MA, Moore JE, Stirman SW, et al. Towards a comprehensive model for understanding adaptations' impact: the model for adaptation design and impact (MADI). *Implementation Science* 2020;15(1):1-15.
15. Miller CJ, Barnett ML, Baumann AA, et al. The FRAME-IS: a framework for documenting modifications to implementation strategies in healthcare. *Implementation Science* 2021;16(1):1-12.
16. Leijten P, Melendez-Torres G, Knerr W, et al. Transported versus homegrown parenting interventions for reducing disruptive child behavior: A multilevel meta-regression study. *Journal of the American Academy of Child & Adolescent Psychiatry* 2016;55(7):610-17.
17. Hawkins J, Madden K, Fletcher A, et al. Development of a framework for the co-production and prototyping of public health interventions. *BMC Public Health* 2017;17(1):1-11.

For peer review only

18. Oliver K, Kothari A, Mays N. The dark side of coproduction: do the costs outweigh the benefits for health research? *Health Research Policy and Systems* 2019;17(1):1-10.
19. Yoong SL, Bolsewicz K, Grady A, et al. Adaptation of public health initiatives: expert views on current guidance and opportunities to advance their application and benefit. *Health Education Research* 2020;35(4):243-57.
20. Movsisyan A, Arnold L, Copeland L, et al. Adapting evidence-informed population health interventions for new contexts: a scoping review of current practice. *Health research policy and systems* 2021;19(1):1-19.
21. Campbell M, Moore G, Evans RE, et al. ADAPT study: adaptation of evidence-informed complex population health interventions for implementation and/or re-evaluation in new contexts: protocol for a Delphi consensus exercise to develop guidance. *BMJ open* 2020;10(7):e038965.
22. Boblin SL, Ireland S, Kirkpatrick H, et al. Using Stake's Qualitative Case Study Approach to Explore Implementation of Evidence-Based Practice. *Qualitative Health Research* 2013;23(9):1267-75. doi: 10.1177/1049732313502128
23. Crowe S, Cresswell K, Robertson A, et al. The case study approach. *BMC medical research methodology* 2011;11:100-00. doi: 10.1186/1471-2288-11-100
24. Yin RK. Enhancing the quality of case studies in health services research. *Health services research* 1999;34(5 Pt 2):1209-24.
25. Gale NK, Heath G, Cameron E, et al. Using the framework method for the analysis of qualitative data in multi-disciplinary health research. *BMC medical research methodology* 2013;13(1):1-8.
26. Aarons GA, Sklar M, Mustanski B, et al. "Scaling-out" evidence-based interventions to new populations or new health care delivery systems. *Implementation Science* 2017;12(1):111.
27. Tremblay M-C, Martin DH, McComber AM, et al. Understanding community-based participatory research through a social movement framework: a case study of the Kahnawake Schools Diabetes Prevention Project. *BMC public Health* 2018;18(1):487.
28. Glasgow RE, Vogt TM, Boles SM. Evaluating the public health impact of health promotion interventions: the RE-AIM framework. *American journal of public health* 1999;89(9):1322-27.
29. Löfholm CA, Brännström L, Olsson M, et al. Treatment ‐ as ‐ usual in effectiveness studies: What is it and does it matter? *International journal of social Welfare* 2013;22(1):25-34.
30. Howard MO, McMillen CJ, Pollio DE. Teaching evidence-based practice: Toward a new paradigm for social work education. *Research on Social Work Practice* 2003;13(2):234-59.
31. Regehr C, Stern S, Shlonsky A. Operationalizing evidence-based practice: The development of an institute for evidence-based social work. *Research on Social Work Practice* 2007;17(3):408-16.
32. Marsiglia FF, Booth JM. Cultural adaptation of interventions in real practice settings. *Research on social work practice* 2015;25(4):423-32.
33. Bonell C, Prost A, Melendez-Torres G, et al. Will it work here? A realist approach to local decisions about implementing interventions evaluated as effective elsewhere. *J Epidemiol Community Health* 2021;75(1):46-50.
34. Fenwick E, Steuten L, Knies S, et al. Value of information analysis for research decisions—an introduction: report 1 of the ISPOR Value of Information Analysis Emerging Good Practices Task Force. *Value in health* 2020;23(2):139-50.

For peer review only

35. Eldridge SM, Lancaster GA, Campbell MJ, et al. Defining feasibility and pilot studies in preparation for randomised controlled trials: development of a conceptual framework. *PloS one* 2016;11(3):e0150205.
36. Tuffaha HW, Roberts S, Chaboyer W, et al. Cost-effectiveness and value of information analysis of nutritional support for preventing pressure ulcers in high-risk patients: implement now, research later. *Applied health economics and health policy* 2015;13(2):167-79.
37. Chu J, Leino A. Advancement in the maturing science of cultural adaptations of evidence-based interventions. *Journal of Consulting and Clinical Psychology* 2017;85(1):45.
38. Moore G, Campbell, M., Copeland, L., Craig, P., Movsisyan, A., Hoddinott, P., Littlecott, H., O'Cathain, A., Pfadenhauer, L., Rehfuess, E., Segrott, J., Hawe,P., Kee, F., Couturiaux, D., Hallingberg, B., Evans, R. Adaptation of interventions for implementation and/or re-evaluation in new contexts: The ADAPT guidance (v1.0). 2020. (accessed 15.4.21).
39. Barrera Jr M, Castro FG, Strycker LA, et al. Cultural adaptations of behavioral health interventions: a progress report. *Journal of consulting and clinical psychology* 2013;81(2):196.
40. Richard L, Torres S, Tremblay M-C, et al. An analysis of the adaptability of a professional development program in public health: results from the ALPS Study. *BMC Health Services Research* 2015;15(1):1-13.
41. Rabin BA, McCreight M, Battaglia C, et al. Systematic, multimethod assessment of adaptations across four diverse health systems interventions. *Frontiers in public health* 2018;6:102.

For peer review only

Appendix A

The Adapt Study:
Development of guidance for funders, researchers, policy-makers and practitioners

Interview Schedule: Researchers

For peer review only

1. **Recording**
    1. Check with the participant that you can record the interview and switch on the recorder
       N.B. For telephone interviews it is important that the recorder is switched on before consent is taken to ensure that we have a record of consent. Ensure that they have received the paper copy of the consent form.

2. **Consent**
    1. Ensure the participant has received and read the information sheet.
    2. Ensure the participant has the opportunity to answer any questions they have about the study.
    3. Take the participant through the consent form and explain each item. Ask the participant to initial each item and sign the form.
    4. Counter-sign the consent form.

3. **Context of Study**

1. Introduce the study. Depending on the professional identity of the participant different levels of explanation will be required on what is adaptation.
2. Prior to the interview select compile an overview of the intervention:
    i. Outcomes; type; target population; activities; theory of change; implementation.
    ii. The context in which the intervention was originally developed/evaluated.
    iii. Evaluation in the original context.
    iv. The context the intervention was adapted to and/or re-evaluated in
    v. Adaptation and evaluation in the new context(s).

For peer review only

4. Questions

Code

1. Domain of question
    i. Question to ask participant
        1. *Prompts / follow-up questions*

1. Participant Details
    i. Can you tell me about yourself?
        1. *Current role? Previous roles? Interest in this area?*

2. What is adaptation?
   Our study is considering how best to adapt or change an intervention so that in can be used in a new context, for example a different country or

with a different group of people. It might include adapting intervention components, implementation or the context.

i. What does the term adaptation mean to you?
ii. Why do you think intervention adaptations are undertaken?
iii. What, if anything, do you think might need to be adapted for an intervention to be used in a new context? What, if anything, do you think should stay the same?
iv. Are there any other terms you might use when thinking about 'adaptation'? Can you describe them?

2. Confirm our Knowledge of Intervention.

We are now going to talk about the intervention you were involved in adapting and / or re-evaluating in a new context (*NB. Participants might not use the term context. They might refer to country, setting etc. Use this terminology as context may seem abstract).*

Summarise what we know about the intervention from the publications etc and confirm if this is broadly correct and if the participant has any additional information or corrections.

3. Deciding on Adaptation

i. What was your involvement with the intervention?
ii. Why was this specific intervention chosen to deliver in the new context?
    1. *Feasible, acceptable, similarity of contexts?*
iii. Was there any flexibility / adaptability built into the original intervention? If so, what was it and why? If not, why not?
    1. *What does fidelity look like in the original form of the intervention?*
iv. Can you summarise the additional changes, if any, you made to the intervention so that it could be delivered in the new context?
v. How did you decide on what would be changed and what would stay the same?
    1. *Who decided?*
    2. *Was there consensus or disagreement? How was this resolved?*
vi. Did you undertaken any other changes that weren't directly related to the intervention? For example did you make any changes to the setting/context? *(Explore changes beyond intervention components and discuss contextual changes if mentioned. Question will need to be amended according to how participant discusses context)*

vii. Were there any changes that you planned to undertake and did not in practice?
viii. Were there any changes that you did not plan for but happened in practice?

4. Process for Undertaking Adaptations
I understand that you undertook the following steps when adapting the intervention so that it could be delivered in the new context (outline process from study reports). Is this correct? Is there anything we have missed out?
    i. How did you decide on this process?
        1. *Who decided?*
        2. *Was there consensus or disagreement? How was this resolved?*
    ii. Was any guidance used to inform this decision-making?
        1. *Which ones? How were they used? What are your reflections on them?*
    iii. Were there any differences between the process you intended to follow and the actual processes undertaken? If so, why?

5. Deciding on re-evaluation (*Wording and focus modified to evaluation that has been conducted)*
I understand that the intervention was re-evaluated in the new context via a pilot trial/RCT/process evaluation etc. and was found to be effective/ineffective/feasible etc. Is this correct?
    i. How did you decide upon the particular approach to re-evaluation?
        1. *Who decided?*
        2. *Was there consensus or disagreement? How was this resolved?*
    ii. Was any guidance use when deciding on the re-evaluation study design?
        2. *Which ones? How were they used? What are your reflections on them?*
    iii. In practice were there any differences between the intended approach to re-evaluation and actual re-evaluation undertaken? If so, why?
    iv. How would you explain the outcome of the evaluation?
        a. *Differences in study design?*
        b. *Contexts similar/dissimilar?*
        c. *Intervention suitable/unsuitable?*

6. Overall reflection on adaptation and re-evaluation

For peer review only

i. What advice would you give to a researcher who was starting out with a similar study to the one you described today?
    1. *What, if anything, was particularly helpful about the way you approached the adaptation and re-evaluation?*
    2. *What, if anything, was particularly unhelpful? What would you do differently in future?*

7. Reporting of adaptation
    i. How was it decided how and where to report the intervention adaptation and evaluation findings?
        1. *Who decided?*
        2. *Was there consensus or disagreement? How was this resolved?*
    ii. What influenced this decision (e.g. worked examples, guidance)?

8. Adaptation guidance
    i. What are your views on having guidance to support researchers/policy-makers/practitioners in undertaking adaptation and/or re-evaluation?
    ii. What would useful guidance on intervention adaptation and/or re-evaluation look like to you?

9. **Closure and Dissemination**

- Thank the participant for their time.
- Explain what will happen with their data next (i.e. will be transferred to secure network server and anonymised)
- Explain what will happen next in the study (i.e. DELPHI study). Ask if we can retain their details to make future contact to potentially invite them to participate in the study. Emphasise that their name will be added to the list as a relevant stakeholder in the field and not because they have participated in the qualitative study – the interviewer will anonymise the data so the participant is not known to the rest of the study team.
- Ask if we can retain their details to make future contact in regard to dissemination (e.g. email list to circulate issued guidance)

For peer review only

The Adapt Study:
Development of guidance for funders, researchers, policy-makers and practitioners

Interview Schedule: Journal Editors / Reviewers

For peer review only

1. **Recording**
    0. Check with the participant that you can record the interview and switch on the recorder

2. **Consent**
    0. Ensure the participant has received and read the information sheet.
    1. Ensure the participant has the opportunity to answer any questions they have about the study.
    2. Ensure the participant has received the consent form and returned a signed copy.
    3. Counter-sign the consent form.

3. **Context of Study**
    0. Introduce the study. Depending on the journal (e.g. generic public health or specialist implementation/adaptation) different levels of explanation will be required on what is adaptation.
    1. Prior to the interview select a couple of examples of adaptation from the journal that you could discuss as concrete examples if required.

4. Questions

Code

0. Domain of question
    i. Question to ask participant
        0. Prompts / follow-up questions

1. Journal and Readership
    i. Can you please tell me about your role at the journal?
    ii. Can you tell me about the remit of the journal and its readership (e.g. discipline, methodological focus)?
        0. How might its remit and readership compare with other journals within the discipline / other inter-disciplinary journals?
        1. Do you think studies reporting adaptations or re-evaluation is a priority for the journal? Why / why not?

2. Decision Making and Assessment Criteria
    i. What is the general process for making decisions about what to publish in the journal (e.g. peer review, editorial recourse)?

ii. How do you make decisions about what to publish?
   0. *Is there generic assessment criteria?*
   1. *Is there specific assessment criteria by study design etc.?*

iii. Does the journal provide reporting/publishing guidance for authors? If so, could you summarize?

3. Interventions Reporting Adaptations and Re-evaluation
   i. If possible, could you outline any key examples of studies published in the journal that report adaptations and/or re-evaluations in new contexts?
      0. *How is adaptation defined in these studies? What do you think about these definitions?*
      1. *What are the types of interventions and outcomes presented?*
      2. *What types adaptations are presented? (Adaptation to components, implementation and/or context)*
      3. *What methodologies were presented??*
      4. *If interventions were being re-evaluated in the new context, what approaches to re-evaluation were presented? How were these justified/explained?*

   ii. Does the journal set any criteria or provide guidance on how to assess either the conduct or reporting of adaptations and/or re-evaluation?
      0. *If so how are these criteria/guidance used by reviewers / how do you use them?*
      1. *What are the strengths and limitations of these criteria/guidance?*
      2. *If there is no criteria/guidance how are decisions made about whether to publish an adaptation and/or re-evaluation study? (N.B. Earlier question on general decision-making, and this is checking more specifically about adaptation studies)*

   iii. Editors: How would you describe the nature and quality of feedback that reviewers provide for adaption and/or re-evaluation studies?
      0. *Are these any common areas of consistency and disagreement?*

   iv. Based on your experience of reviewing/publishing adaptation and/or re-evaluation studies are there key strengths you have observed across studies? (e.g. tend to be a comprehensive adaptation process)

   v. Are there key limitations you have observed across studies? (e.g. poor description of rationale for adaptations)

   vi. Are there key recommendations you have to strengthen studies that undertake adaptations and/or re-evaluation?

For peer review only

4. Adaptation and Re-valuation Guidance
    i. Do you think guidance might support the process of deciding whether to publish adaptation and/or re-evaluation study? Why and how?
    ii. What would useful guidance on intervention adaptation and/or re-evaluation look like to you?

5. Closure and Dissemination

0. Thank the participant for their time.
1. Explain what will happen with their data next (i.e. will be transferred to secure network server and anonymised)
2. Explain what will happen next in the study (i.e. DELPHI study). Ask if we can retain their details to make future contact to potentially invite them to participate in the study. Emphasise that their name will be added to the list as a relevant stakeholder in the field and not because they have participated in the qualitative study – the interviewer will anonymise the data so the participant is not known to the rest of the study team.
3. Ask if we can retain their details to make future contact in regard to dissemination (e.g. email list to circulate issued guidance)

For peer review only

The Adapt Study:
Development of guidance for funders, researchers, policy-makers and practitioners

Interview Schedule: Funders

For peer review only

1. **Recording**
    1. Check with the participant that you can record the interview and switch on the recorder
       N.B. For telephone interviews it is important that the recorder is switched on before consent is taken to ensure that we have a record of consent. Ensure that they have received the paper copy of the consent form.

2. **Consent**
    1. Ensure the participant has received and read the information sheet.
    2. Ensure the participant has the opportunity to answer any questions he has about the study.
    3. Take the participant through the consent form and explain each item. Ask the participant to initial each item and sign the form.
    4. Counter-sign the consent form.

3. Questions

1. Can you please tell me about your role on the funding panel?

2. Funding Panel
    i. Can you tell me about the remit of the funding panel (e.g. types of study, outcome focus)?
    ii. Can you tell me about the membership of the funding panel (e.g. expertise)?
    iii. How do you think the funding panel's remit and expertise fit with the wider funding context, both nationally and internationally?

3. Decision Making and Assessment Criteria
    i. What is the process for making decisions on the panel?
        1. Prioritisation panel?
        2. Scientific panel?
    ii. How do you make decisions about what to fund?
        1. Is there generic assessment criteria?
        2. Is there specific assessment criteria by study design etc.?

4. Interventions Proposing Adaptations and Re-evaluation

For peer review only

i. Does you panel have a working definition of adaptation? If so, what is it?
    1. Is there consensus/disagreement on the panel over what adaptation means?
    2. Do you use other terms to describe adaptation, and why?

ii. Does your panel set any criteria or provide guidance for applicants proposing to conduct adaptations (e.g. 6SQUiD for development)?
    1. If so how are these criteria/guidance used by applicants?
    2. How useful are these criteria/guidance to the funding panel?

iii. Can you tell me about your experience of funding studies that include proposed adaptations:
    1. What were the types, theories and outcomes of interventions proposed?
    2. What types of adaptations are proposed?
    3. What types of studies are proposed (e.g. development, outcome evaluation, and implementation)?
    4. How to studies define and analyse context?
    5. Do you have reflections on the strengths and limitations of proposed adaptions?

iv. How does the panel decide about the appropriateness of proposed adaptations?
    1. Are there key areas of consensus?
    2. Are there key areas of disagreement?

v. How does the panel decide about the appropriateness of proposed re-evaluation?
    1. Are there key areas of consensus?
    2. Are there key areas of disagreement?

vi. Are there ways in which you think proposed adaptations and / or re-evaluation studies could be strengthened?

5. Reporting of Adaptations in Proposals

i. Does your panel set any criteria or provide guidance for applicants on the reporting and dissemination of adaptation studies?
    1. If so how are these criteria/guidance used by applicants?
    2. How useful are these criteria/guidance to the funding panel?

6. Adaptation and Re-valuation Guidance

i. Do you think guidance might support the process of deciding when to fund an adaptation and/or re-evaluation study? Why and how?

ii. What would useful guidance on intervention adaptation and/or re-evaluation look like to you?

For peer review only

4. **Closure and Dissemination**
    1. Thank the participant for their time.
    2. Explain what will happen with their data next (i.e. will be transferred to secure network server and anonymised)
    3. Explain what will happen next in the study (i.e. DELPHI study)
        i. Check with the study team if we would like to invite this participant to complete the DELPHI, and if so ask if they would like to be contacted about participation.
    4. Ask if we can retain their details to make future contact in regard to dissemination (e.g. email list to circulate issued guidance)

For peer review only

Standards for Reporting Qualitative Research (SRQR)*

http://www.equator-network.org/reporting-guidelines/srqr/

Title and abstract

	Page/line no(s).
**Title** - Concise description of the nature and topic of the study Identifying the study as qualitative or indicating the approach (e.g., ethnography, grounded theory) or data collection methods (e.g., interview, focus group) is recommended	1
**Abstract** - Summary of key elements of the study using the abstract format of the intended publication; typically includes background, purpose, methods, results, and conclusions	2

Introduction

**Problem formulation** - Description and significance of the problem/phenomenon studied; review of relevant theory and empirical work; problem statement	3
**Purpose or research question** - Purpose of the study and specific objectives or questions	3

Methods

**Qualitative approach and research paradigm** - Qualitative approach (e.g., ethnography, grounded theory, case study, phenomenology, narrative research) and guiding theory if appropriate; identifying the research paradigm (e.g., postpositivist, constructivist/ interpretivist) is also recommended; rationale**	P 4 line120
**Researcher characteristics and reflexivity** - Researchers' characteristics that may influence the research, including personal attributes, qualifications/experience, relationship with participants, assumptions, and/or presuppositions; potential or actual interaction between researchers' characteristics and the research questions, approach, methods, results, and/or transferability	P5 line 179
**Context** - Setting/site and salient contextual factors; rationale**	P 3 and 4
**Sampling strategy** - How and why research participants, documents, or events were selected; criteria for deciding when no further sampling was necessary (e.g., sampling saturation); rationale**	P4 line 126
**Ethical issues pertaining to human subjects** - Documentation of approval by an appropriate ethics review board and participant consent, or explanation for lack thereof; other confidentiality and data security issues	P4 line 118
**Data collection methods** - Types of data collected; details of data collection procedures including (as appropriate) start and stop dates of data collection and analysis, iterative process, triangulation of sources/methods, and modification of procedures in response to evolving study findings; rationale**	P4 Line 150

For peer review only - http://bmjopen.bmj.com/site/about/guidelines.xhtml

**Data collection instruments and technologies** - Description of instruments (e.g., interview guides, questionnaires) and devices (e.g., audio recorders) used for data collection; if/how the instrument(s) changed over the course of the study	P4 Line 150
**Units of study** - Number and relevant characteristics of participants, documents, or events included in the study; level of participation (could be reported in results)	P4 line 140
**Data processing** - Methods for processing data prior to and during analysis, including transcription, data entry, data management and security, verification of data integrity, data coding, and anonymization/de-identification of excerpts	P5 line 159
**Data analysis** - Process by which inferences, themes, etc., were identified and developed, including the researchers involved in data analysis; usually references a specific paradigm or approach; rationale**	P5 line 159
**Techniques to enhance trustworthiness** - Techniques to enhance trustworthiness and credibility of data analysis (e.g., member checking, audit trail, triangulation); rationale**	P5 Line 174

Results/findings

**Synthesis and interpretation** - Main findings (e.g., interpretations, inferences, and themes); might include development of a theory or model, or integration with prior research or theory	P8-13
**Links to empirical data** - Evidence (e.g., quotes, field notes, text excerpts, photographs) to substantiate analytic findings	P8-13

Discussion

**Integration with prior work, implications, transferability, and contribution(s) to the field -** Short summary of main findings; explanation of how findings and conclusions connect to, support, elaborate on, or challenge conclusions of earlier scholarship; discussion of scope of application/generalizability; identification of unique contribution(s) to scholarship in a discipline or field	P13-14
**Limitations** - Trustworthiness and limitations of findings	P14 Line 461

Other

**Conflicts of interest** - Potential sources of influence or perceived influence on study conduct and conclusions; how these were managed	P 15 Line 500
**Funding** - Sources of funding and other support; role of funders in data collection, interpretation, and reporting	P15 Line 488

*The authors created the SRQR by searching the literature to identify guidelines, reporting standards, and critical appraisal criteria for qualitative research; reviewing the reference lists of retrieved sources; and contacting experts to gain feedback. The SRQR aims to improve the transparency of all aspects of qualitative research by providing clear standards for reporting qualitative research.

For peer review only

**The rationale should briefly discuss the justification for choosing that theory, approach, method, or technique rather than other options available, the assumptions and limitations implicit in those choices, and how those choices influence study conclusions and transferability. As appropriate, the rationale for several items might be discussed together.

Reference:

O'Brien BC, Harris IB, Beckman TJ, Reed DA, Cook DA. **Standards for reporting qualitative research: a synthesis of recommendations.** *Academic Medicine*, Vol. 89, No. 9 / Sept 2014 DOI: 10.1097/ACM.0000000000000388

For peer review only

For peer review only - http://bmjopen.bmj.com/site/about/guidelines.xhtml
